# The importance of older patients’ experiences with care delivery for their quality of life after hospitalization

**DOI:** 10.1186/s12913-015-0982-1

**Published:** 2015-08-08

**Authors:** Jacqueline M. Hartgerink, Jane M. Cramm, Ton J. Bakker, Johan P. Mackenbach, Anna P. Nieboer

**Affiliations:** Institute of Health Policy & Management (iBMG), Erasmus University, Rotterdam, The Netherlands; Argos Zorggroep, Schiedam, The Netherlands; Department of Public Health, Erasmus MC, Rotterdam, The Netherlands

## Abstract

**Background:**

Older patients’ experiences with care delivery may be important for their quality of life over time. Evidence is however lacking. Therefore, this study aims to identify the longitudinal relationship between older patients’ experiences with hospital care, perceived quality of integrated care and quality of life after hospitalization.

**Methods:**

Our longitudinal research was based on a pilot study of older people who had recently been admitted to a hospital. In the pilot study, all patients (≥65 years of age) who were admitted to the Vlietland hospital between June and October 2010 were asked to participate, which led to the inclusion of 500 older patients at baseline. A total of 291 patients (58 % response rate) were interviewed 3 months after admission. Measures included quality of life, patients’ perceptions of quality of integrated care delivery and patients’ experiences with hospital care. We used descriptive statistics, correlations, and multilevel analyses.

**Results:**

Being married (*p* ≤ 0.05), patients’ experiences with hospital care, perceived quality of integrated care delivery (both *p* ≤ 0.01), and quality of life within 48 h of hospital admission (*p* ≤ 0.001) significantly correlated with quality of life 3 months after hospital admission. After controlling for background characteristics, multilevel analysis indicated a longitudinal relationship between patients’ experiences with hospital care (*p* ≤ 0.05), perceived quality of integrated care delivery (*p* ≤ 0.01) and patients’ quality of life 3 months after hospitalization.

**Conclusions:**

This study found a longitudinal relationship between patients’ perceived quality of integrated care delivery, experiences with hospital care and quality of life of older patients after hospitalization. These results underscore the importance of enhancing older patients’ experiences with care delivery.

**Electronic supplementary material:**

The online version of this article (doi:10.1186/s12913-015-0982-1) contains supplementary material, which is available to authorized users.

## Background

With the aging population healthcare professionals are increasingly dealing with older patients suffering from multiple chronic diseases. This poses challenges for the complex coordination of tasks performed during care delivery [1]. Their intense use of health services puts older patients at greater risk of receiving fragmented or poor-quality care [2, 3]. Once admitted to the hospital, older patients are at an increased risk for poor outcomes such as readmission, increased length of stay, functional decline, iatrogenic complications, and nursing home placement [4]. Schwarz [5] found a 33 % rate of readmission within 3 months for older patients, which is consistent with other studies of readmission rates among these patients. Sager and colleagues [6] found that the ability to perform one or more activities of daily living had declined in 32 % of older patients at the time of discharge. Of functional independent individuals of 65 years and older admitted to the hospital from their home for acute illness, 75 % experienced adverse events after hospitalization, including 15 % who were discharged to nursing homes [7]. So for many older patients, hospitalization is followed by an often irreversible decline in functional status and quality of life [8].

The organization and delivery of hospital care is often fragmented, uncoordinated, and duplicated, which negatively affects patients’ quality of life [3, 9–11]. Since a substantial number of older patients suffer from a mixture of problems in multiple life domains, protecting their quality of life not only concerns physical health, but also involves social and psychological well-being [12]. Vulnerable older patients have complicated and on-going needs, experience difficulties in everyday living, require a mix of services delivered sequentially or simultaneously by multiple providers and receive both cure and care [13]. The provision of healthcare, social services and related services at the right time and place to such older patients is of high importance. Problems typically include difficulties with obtaining needs assessments, putting together comprehensive service packages, coordinating multiple providers and services, ensuring continuity, and monitoring health and functional status [14]. To solve many of these problems, the literature strongly suggests that holistic and personalized integrated care delivery encompassing the total care process is required [3, 15, 16]. This approach enhances quality of care and provides better levels of service; that is, one that is more sensitive to the personal circumstances and wishes of the individual patient [13]. Through the integration of interrelated care delivery components (e.g. case management, support systems, multidisciplinary teamwork, treatment plans), all the activities and information about the needs of the patient are coordinated, placing the older patient in the centre of the care process [17–19]. Integrated care delivery has shown to improve quality of care due to patient involvement in planning of care, better patient education, more staff time with patients and improved communication between professionals [20]. As a result, length of stay in the hospital decreased, and complications were reduced [21, 22].

Since the healthcare delivery system is known to be a major factor contributing to health and quality of life outcomes for hospitalized older adults [9, 10], patients’ experiences with care may be beneficial to their quality of life. Several studies indeed have demonstrated a positive association between patients’ experiences with hospital care and patients’ functional health [23–25], self-perceived health and emotional health [26, 27]. Evidence has shown that when experiences with care are less satisfactory this is associated with non-compliance with treatment, return appointments, and a poor understanding and retention of medical information [28]. As such patients’ experiences with hospital care may be directly related to improvements in quality of life, and healthcare outcomes [28–31]. As such we expect that experiences with care delivery as well as perceptions of integrated care delivery are positively related to older patients’ quality of life. Evidence supporting this expectation is however lacking. Therefore, this study aims to identify the longitudinal relationship between patients’ experiences with hospital care, perceived quality of integrated care and quality of life among older patients after hospitalization.

## Methods

### Setting and design

The current study was conducted in 2010 among older patients who were admitted to a hospital in the context of the Prevention and Reactivation Care Program [32], which was designed to prevent loss of function in older patients due to hospitalization and targeted older hospital patients (≥65 years of age) who were vulnerable to loss of function after hospital discharge. This research is based on the pilot study of 500 patients (≥65 years old) prior to implementation of the Prevention and Reactivation Care Program. The results of the pilot study have been used to identify possible practical implementation problems in preparation for the main evaluation study and serve as a base for power calculations for the main study.

A total of 1026 patients admitted to the Vlietland hospital in The Netherlands between June and October 2010 were approached to participate in the study. We excluded patients who refused participation, did not understand the Dutch language, were expected to stay in the hospital for less than 48 h, were unable to answer questions or follow instructions due to cognitive problems (MMSE score lower than 12), or had a life expectancy of less than 3 months. Five hundred agreed to participate and signed an informed consent form (response 49 %). Three months after admission, 173 participants had been lost to follow up, 36 participants had died, and 291 people (response rate 58 %) were interviewed.

The study complies with the rules as stated in the Declaration of Helsinki. The medical ethics committee of the Erasmus Medical Centre, Rotterdam, the Netherlands, approved methods for data collection (protocol number MEC2011-041).

### Questionnaire

Quality of life was assessed with an adjusted version of Cantril’s Self Anchoring Ladder [33]. Within 48 h after hospital admission (T0) and 3 months after hospital admission (T1), respondents were asked to rate their lives on a scale of 0–10 by answering the following questing: Which report mark would you give your life at this moment?

Patients’ perceptions of quality of integrated care delivery were assessed with the 10-item Older Patients’ Assessment of Integrated Care (O-PACIC scale, see Additional file 1: Appendix 1) [34], which was based on the 20-item Patient Assessment of Chronic Illness Care questionnaire (PACIC) [35]. The PACIC is intended to assess the receipt of integrated care according to the principals of the Chronic Care Model. The Chronic Care Model provides an organized multidisciplinary approach to care delivery. The idea of this model is to transition care delivery from reactive to proactive, planned, integrated, holistic and personalized. Given the complex medical, social, and psychological problems of both chronically-ill and older patients they are both in need of an organised multidisciplinary and integrated approach to care delivery [34]. Therefore, improvements in integrated care delivery in hospitals according to the principles of the Chronic Care Model are expected to be beneficial for older patients as well. The PACIC has internationally been used as an instrument to evaluate integrated care delivery among patients with various chronic conditions. For a full overview see a recent publication of Iglesias and colleagues [36] testing all published validation models of the PACIC. With this instrument patients are asked if their care was well organized, if they were given choices about their treatment to think about and if they were helped to make a treatment plan that they could fit in their daily life. Research indeed indicates that these are all important issues to improve outcomes for older patients after hospitalization and reduce poor outcomes such as readmission, functional decline, mortality, nursing home placement, and healthcare costs [5, 6, 37–40]. The O-PACIC was developed and validated as a reliable, feasible instrument to assess older patients’ experiences with integrated care delivery after hospitalization showing strong psychometric properties [34]. The O-PACIC score represents the sum of the participants’ responses divided by 10. Scores ranged from 1 to 5, with higher scores indicating a greater perception of receipt of integrated care delivery during hospital stay. At T1 (3 months after hospital admission) respondents were asked to give their perception on the quality of integrated care delivery. The Cronbach’s alpha coefficient of the scale in this study was 0.75, indicating reliability.

The Hospital Consumer Assessment of Healthcare Providers and Systems (HCAHPS survey, see Additional file 1: Appendix 2) is an 18-item questionnaire which assesses patients’ experiences with hospital care. These 18 items include two global ratings of hospital care delivery and 16 questions that relate to their recent hospital experience. The two global rating items assess patients’ overall rating of the hospital using a 0 (worst hospital possible) to 10 (best hospital possible) rating scale. Willingness to recommend the hospital to friends and family is assessed by using a 4-point scale ranging from *definitely no* to *definitely yes*. Of the 16 experience questions two items are answered with a simple *yes* or *no*. These two items assess patients’ experiences regarding cleanliness of hospital environment and quietness of hospital environment. Patients are asked to rate their level of agreement to the remaining 14 items on a 4-point scale ranging from *never* to *always.* These 14 questions are used to construct the following 6 subscales: communication with nurses (3 questions), communication with doctors (three questions), responsiveness of hospital staff (two questions), pain management (two questions), communication about medicines (two questions), and discharge information (two questions) [41, 42]. The standardized HCAHPS score was the mean of the participants’ responses on the subscales, individual items and global ratings. Scores ranged from 0 to 2, with higher scores indicating better experiences with the received hospital care. At T1 (3 months after hospital admission) respondents were asked to assess their hospital experience 3 months earlier. The Cronbach’s alpha coefficient for this instrument was 0.87, indicating good reliability.

We further asked participants for age, gender, marital status, education level, length of hospital stay, general health, and cognitive and physical functioning. Education was assessed on seven levels ranging from (1) no school or some primary education (6 years of education or less) to (7) university degree (18 years of education or more). In our analyses, we dichotomized this into (1) low educational level (followed school after primary education, but without a diploma or less), and (0) followed school after primary education with diploma or higher. The length of hospital stay was used as a proxy for severity of the patients’ medical problems for which he/she was admitted. The participants’ general health was assessed on a 5-point scale (1 = excellent, 2 = very good, 3 = good, 4 = reasonable, 5 = bad). We dichotomized this into (1) bad health (scores 4 and 5), and (0) good health (scores 1, 2 and 3). Cognitive functioning was assessed with the Mini Mental State Examination (MMSE), which measures cognitive functioning by asking questions about orientation in time and space, short- and middle-term memory, comprehension, and other cognitive dimensions. Scores ranged from 0 to 30, with higher scores indicating higher levels of cognitive functioning. Any score equal or above 25 points (of 30) represents effective cognitive functioning (intact). Below this, scores can indicate severe (≤9 points), moderate (10–20 points), or mild (21–24 points) cognitive functioning losses [43, 44]. Physical functioning was assessed using the Katz Index of independence in activities of daily living [45, 46], which ranks an individuals’ ability to perform six functions: bathe, dress, use the toilet, transfer, remain continent, and feed oneself. Scores of no (1) or yes (0) indicate (in)dependence in each function, with 6 is full physical function, 4 is moderate, and equal or below 2 is severe physical function impairment.

### Statistical analysis

Descriptive statistics were used to analyze patients’ age, gender, marital status, education level, length of hospital stay and health. Correlation analysis was used to investigate the relationship between the background characteristics, patients’ general health, cognitive functioning, physical functioning, experiences with hospital care, perceived quality of integrated care delivery, and quality of life. We employed a random-effects multilevel model to investigate the relationship between older patients’ perception of the quality of integrated care delivery, experiences with hospital care and quality of life over time. Background characteristics and significant univariate associations with quality of life at T1 (3 months after hospital admission) were included in the multilevel analyses. A significance level of 0.05 was used for all statistical tests. Data were analyzed using the SPSS software package (ver. 18.0 for Windows; SPSS Inc., Chicago, IL, USA).

## Results

Descriptive statistics are displayed in Table 1. Respondents’ mean age was 75.9 (±7.2; range 65–95); slightly more were female (55.2 %). Just over half were married/ living together (56.9 %); the others were single, widowed or divorced (43.1 %). Of the respondents, 34.7 % had a low educational level. On average, patients stayed 6.6 days (±6.7) in the hospital and 40.5 % rated their health as poor. At T0 (within 48 h after hospital admission), the mean score for cognitive functioning was 26.2 (±4.3), indicating intact cognitive functioning. The mean score for physical functioning was 3.9 (±2.0), indicating moderate physical function impairment. The mean score for quality of life at T0 (within 48 h after hospital admission) was 7.2 (±1.4). At T1 (3 months after hospital admission), the mean score for quality of life was 7.4 (±1.3). The respondents who were lost to follow-up between T0 and T1 did not differ significantly in their score for quality of life at T0 (*p* = 0.45). On 1–5 scale, the mean quality of integrated care delivery as measured with the O-PACIC was 1.8 (±0.6). The respondents reported a mean overall experience with hospital care of 1.2 (±0.4) as measured on the 0 to 2 HCAHPS scale.

**Table 1:** Descriptive statistics

Demographic characteristics	Range	% or mean (SD)
Quality of life T0	1-10	7.2 (1.4)
Gender (female)		55.2 %
Age	65-95	75.9 (7.2)
Marital status (married)		56.9 %
Educational level (low)		34.7 %
Length of hospital stay	0-65	6.6 (6.7)
Health (poor)		40.5 %
Cognitive functioning	0-30	26.2 (4.3)
Physical functioning		3.9 (2.0)
Experiences with hospital care		1.2 (0.4)
Perception of integrated care delivery		1.8 (0.6)
Quality of life T1		7.4 (1.3)

*SD* standard deviation; *T0* within 48 h after hospitalization; *T1* 3 months after hospitalization

### Experiences with hospital care, integrated care delivery and quality of life

Correlation analysis revealed that being married (*r* = 0.14; *p* ≤ 0.05), experiences with hospital care at T1 (3 months after hospitalization; *r* = 0.17; *p* ≤ 0.01), quality of integrated care delivery at T1 (3 months after hospitalization; *r* = 0.18; *p* ≤ 0.01) and quality of life at T0 (within 48 h after hospital admission) (*r* = 0.43; *p* ≤ 0.001) were positively associated with quality of life at T1 (3 months after hospitalization). Poor health at T0 (within 48 h after hospital admission; *r* = −0.27; *p* ≤ 0.001) showed a negative correlation with quality of life at T1 (3 months after hospitalization) (Table 2).

**Table 2:** Correlations between background characteristics, integrated care delivery, satisfaction with hospital care and quality of life

	Quality of life T1	*n*
Quality of life T0	0.43***	286
Gender (female)	0.02	289
Age	−0.04	288
Marital status (married)	0.14*	289
Educational level (low)	−0.10	289
Length of hospital stay	−0.02	289
Health (poor)	−0.27***	289
Cognitive functioning	0.11	289
Physical functioning	0.09	289
Experiences with hospital care	0.17**	287
Perception of integrated care delivery	0.18**	279

*T0* within 48 h after hospitalization; *T1* 3 months after hospitalization

*p ≤ 0.05; **p ≤ 0.01; ***p ≤ 0.001 (two-tailed)

The results of multilevel analyses are displayed in Table 3. These analyses showed that experiences with hospital care (*p* ≤ 0.05) and quality of integrated care delivery (*p* ≤ 0.01) were positively related to patients’ quality of life at T1 (3 months after hospitalization).

**Table 3:** Quality of life predictors at T1 as assessed by multilevel regression analyses (random intercepts model) (*n* = 264)

	B	SE	ß	SE
Constant	3.72	0.99	7.36	0.08
Quality of life T0	0.36***	0.06	0.51***	0.08
Gender (female)	0.32*	0.15	0.16	0.08
Age	−0.00	0.01	0.00	0.08
Marital status (married)	0.29	0.16	0.14	0.08
Educational level (low)	−0.24	0.16	−0.11	0.08
Length of hospital stay	0.00	0.02	0.00	0.22
Health (poor)	−0.50***	0.15	−0.45***	0.07
Experiences with hospital care	0.38*	0.18	0.15*	0.07
Perception of integrated care delivery	0.31**	0.12	0.19**	0.07

*T0* within 48 h after hospitalization; *T1* 3 months after hospitalization; *B* unstandardized results; *ß* standardized results; *SE* standard error; multilevel analyses included respondents who filled in questionnaire at T0 and T1 only (*n* = 291); listwise deletion of missing cases resulted in 264 cases for the multilevel regression analyses

*p ≤ 0.05; **p ≤ 0.01; ***p ≤ 0.001 (two-tailed)

## Discussion

This study aimed to identify the longitudinal relationship between patients’ experiences with hospital care, perceived quality of integrated care and quality of life among older patients after hospitalization. Our results showed that quality of life 3 months after hospitalization was indeed related to patients’ experiences with hospital care and quality of integrated care delivery over time. This implies that older patients who are more satisfied about the received hospital care and experience higher levels of integrated care delivery are those with a higher quality of life 3 months after hospitalization. Such results align with those of previous studies, which have found that integrated care delivery had a positive effect on quality of life [1, 47].

Healthcare improvement programs at hospitals usually focus on isolated interventions, such as medication supply or multidisciplinary cooperation, rather than on the total care process of the patient [15]. Integrated care programs have begun to receive support as approaches to reduce fragmentation of care and to achieve improved results for patient outcomes [17]. Our findings are based on a pilot study conducted in 2010 among older people who had recently been admitted to a Dutch hospital in the context of the Prevention and Reactivation Care Program [32]. This integrated care program supports a multifaceted and multidisciplinary approach to the care of older patients organized around several core components, including screening for vulnerability, early detection and treatment of health problems, case management, and multidisciplinary teamwork. The main goal of the program is to reduce the loss of function among older patients after hospital discharge. The current study shows the importance of assessing the perspective of older patients on the amount of care integration and patients’ experiences with hospital care while evaluating care delivery, as they could be a predictor of the outcomes in quality improvement programs.

The question for most professionals is how to enhance patients’ experiences with (integrated) care delivery while keeping costs low and make efficient use of resources. Professionals often struggle on how to balance between these often competing needs [48]. Interventions that focus on patient education may be a useful addition to the usual integrated care components of case management, support systems, multidisciplinary teamwork, and treatment plans [12, 17]. Shen and colleagues [49] showed that a nurse conducted education program for hospitalized older patients resulted in improved medication knowledge. Since integrated care delivery emphasizes the importance of informed patients that can interact with proactive professional teams [17], education about medication usage and health conditions could be of added value. This could result in more satisfied patients with a better quality of life [50].

The limitations of this study should be considered when interpreting the findings. Firstly, patients were asked to provide a grade for their quality of life within 48 h after hospital admission and 3 months after hospital discharge. No information is available of their quality of life prior to their hospital admission, which is expected to be an additional indicator for quality of life after hospitalization. Secondly, we asked participants to rate the level of integrated care delivery and experiences with hospital care 3 months after they were discharged from the hospital. This retrospective design may have had an effect on how the hospital experience is recalled [51]. Thirdly, although our study showed that patients’ experiences with hospital care had a positive relationship with older patients’ perception of the quality of integrated care delivery, the question then becomes how these concepts are linked. While both instruments—the HCAHPS and O-PACIC—evaluate aspects of experienced quality of hospital care, they both focus on different concepts related to hospital care. The HCAHPS survey evaluates experienced care, mainly focusing on ‘performance and attitude of physicians and nurses’, while the O-PACIC survey evaluates the experienced degree in which care delivery is integrated and coordinated (both in the opinion of patient). Fourthly, the relationship between patients’ experiences with hospital care, perceived quality of integrated care and quality of life among older patients after hospitalization may be dynamic; while better experiences with (integrated) care may improve patients’ quality of life one may also anticipate that poorer quality of life negatively affects patients’ experiences with care delivery. Differences in morbidity and health care needs among patients, may affect their experiences with care delivery. Future research has to further explore the link between experiences with (integrated) care and quality of life among patients. Fifthly, this study included patients’ perceptions only. We did not include objective information concerning clinical status of patients. Although respondents who were lost to follow-up between measurements did not differ significantly in their score for quality of life we do not know if their clinical situation differed. Finally, our study sample consisted of older people who had recently been admitted to the hospital, which limits generalizability of our study findings to e.g. integrated care programs in other healthcare settings or the community.

## Conclusions

We conclude that older patients’ experiences with (integrated) hospital care are important for their quality of life. These results underscore the importance of further studying older patients’ experiences with hospital care.

## Additional file

Additional file 1:
**Appendix: 1 O-PACIC scale.** Appendix 2: HCAHPS survey. (DOCX 15 kb)
