# Supplementary material for: The importance of older patients’ experiences with care delivery for their quality of life after hospitalization
Source: BMC Health Serv Res. 2015 Aug 8;15:311. doi: 10.1186/s12913-015-0982-1 (PMC4529729; doi:10.1186/s12913-015-0982-1)
Supplement: Additional file 1: — Appendix: 1 O-PACIC scale. Appendix 2: HCAHPS survey. (DOCX 15 kb) [file 12913_2015_982_MOESM1_ESM.docx]

**Appendix 1 O-PACIC scale**

|  | ***When I received care....*** |
| --- | --- |
| 1. | ... I was given choices on treatment to think about |
| 2. | ... I was asked to talk about any problems with my medicines or their effects |
| 3. | ... I was given a written list of things I should do to improve my health |
| 4. | ... I was shown how what I did to take care of my illness influenced my condition |
| 5. | ... I was asked to talk about my goals in caring for my illness |
| 6. | ... I was helped to set specific goals to improve my eating or exercise |
| 7. | ... I was encouraged to go to a specific group/class to help me cope with my illness |
| 8. | ... I was helped to make a treatment plan that I could do in my daily life |
| 9. | ... I was asked how my illness affects my life |
| 10. | ... I was contacted after a visit to see how things were going |

**Appendix 2 HCAHPS survey**

| 1. | Communication with nurses: During this hospital stay, how often did nurses treat you with courtesy and respect? |
| --- | --- |
| 2. | Communication with nurses: During this hospital stay, how often did nurses listen carefully to you? |
| 3. | Communication with nurses: During this hospital stay, how often did nurses explain things in a way you could understand? |
| 4. | Communication with doctors: During this hospital stay, how often did doctors treat you with courtesy and respect? |
| 5. | Communication with doctors: During this hospital stay, how often did doctors listen carefully to you? |
| 6. | Communication with doctors: During this hospital stay, how often did doctors explain things in a way you could understand? |
| 7. | Responsiveness of hospital staff: During this hospital stay, after you pressed the call button, how often did you get help as soon as you wanted it? |
| 8. | Responsiveness of hospital staff: How often did you get help in getting to the bathroom or in using a bedpan as soon as you wanted? |
| 9. | Pain management: During this hospital stay, how often was your pain well controlled? |
| 10. | Pain management: During this hospital stay, how often did the hospital staff do everything they could to help you with your pain? |
| 11. | Communication about medicines: Before giving you any new medicine, how often did hospital staff tell you what the medicine was for? |
| 12. | Communication about medicines: Before giving you any new medicine, how often did hospital staff describe possible side effects in a way you could understand? |
| 13. | Cleanliness of hospital environment: During this hospital stay, how often were your room and bathroom kept clean? |
| 14. | Quietness of hospital environment: During this hospital stay, how often was the area around your room quiet at night? |
| 15. | Discharge information: During this hospital stay, did doctors, nurses or other hospital staff talk with you about whether you would have the help you needed when you left the hospital? |
| 16. | Discharge information: During this hospital stay, did you get information in writing about what symptoms or health problems to look out for after you left the hospital? |
| 17 | Overall hospital rating: Using any number from 0 to 10, where 0 is the worst hospital possible and 10 is the best hospital possible, what number would you use to rate this hospital during your stay? |
| 18. | Recommend the hospital: Would you recommend this hospital to your friends and family? |
